# The management correlation between metabolic index, cardiovascular health, and diabetes combined with cardiovascular disease

**DOI:** 10.3389/fendo.2022.1036146

**Published:** 2023-01-27

**Authors:** Yi Zhang, Chao Liu, Yijing Xu, Yanlei Wang, Fang Dai, Honglin Hu, Tian Jiang, Yunxia Lu, Qiu Zhang

**Affiliations:** ^1^ Department of Endocrinology, The First Affiliated Hospital of Anhui Medical University, Hefei, Anhui, China; ^2^ Department of Maternal, Child and Adolescent Health, School of Public Health, Anhui Medical University, Hefei, Anhui, China; ^3^ Department of Biochemistry and Molecular Biology, Anhui Medical University, Hefei, China; ^4^ The Comprehensive Laboratory, School of Basic Medical Science, Anhui Medical University, Hefei, China

**Keywords:** cardiovascular diseases, metabolic index, cardiovascular health (CVH), diabetes, management

## Abstract

**Background:**

Cardiovascular disease (CVD) has become a major cause of morbidity and mortality in patients with type 2 diabetes mellitus (T2DM). Although there is also evidence that multifactorial interventions to control blood glucose, blood pressure, and lipid profiles can reduce macrovascular complications and mortality in patients with T2DM, the link between these risk factors has not been established.

**Methods:**

On 10 December 2018, 1,920 people in four cities in Anhui Province were included. Latent category analysis (LCA) was used to explore the clustering mode of HRBs (health risk behaviors). The primary exposure was HRBs and exercise and diet interventions, and the primary outcome was CVD and other variables, including zMS, triglyceride-glucose index (TyG), TyG-WC (waist circumference), TyG-BMI, TG/HDL, and cardiovascular health (CVH). A multivariable logistic regression model was used to establish the relationship between HRBs, exercise, diet interventions, and CVD. Moderate analysis and mediation moderation analysis were employed by the PROCESS method to explore the relationship between these variables. Sensitivity analysis explored the robustness of the model.

**Results:**

The mean age was 57.10 ± 10.0 years old. Overall, CVD affects approximately 19.9% of all persons with T2DM. Macrovascular complications of T2DM include coronary heart disease, myocardial infarction (MI), cardiac insufficiency, and cerebrovascular disease. Elderly age (*χ* ^2^ = 22.70), no occupation (*χ* ^2^ = 20.97), medium and high socioeconomic status (SES) (*χ* ^2^ = 19.92), higher level of TyG-WC (*χ* ^2^ = 6.60), and higher zMS (*χ* ^2^ = 7.59) were correlated with high CVD. Many metabolic indices have shown a connection with T2DM combined with CVD, and there was a dose−response relationship between HRB co-occurrence and clustering of HRBs and zMS; there was a dose−response relationship between multifactorial intervention and CVH. In the mediation moderation analysis, there was an association between HRB, gender, TyG, TyG-BMI, and CVD. From an intervention management perspective, exercise and no diet intervention were more significant with CVD; moreover, there was an association between intervention management, gender, zMS, TyG-WC, TyG-BMI, TG/HDL, and CVD. Finally, there was an association between sex, CVH, and CVD. Sensitivity analysis demonstrated that our results were robust.

**Conclusions:**

CVD is one of the common complications in patients with type 2 diabetes, and its long-term outcome will have more or less impact on patients. Our findings suggest the potential benefits of scaling up multifactorial and multifaceted interventions to prevent CVD in patients with T2DM.

## 1 Introduction

### 1.1 The prevalence of cardiovascular diseases and influencing factors

Diabetes is a common chronic disease and one of the major diseases affecting people’s lives worldwide (1, 2). In 2021, the International Diabetes Federation stated that the number of people with type 2 diabetes (T2DM) had risen to 597 million (3). T2DM is associated with a range of adverse outcomes, such as hypertension, hyperlipidemia, metabolic abnormalities, and other negative outcomes; among them, T2DM patients also have a significantly increased risk of cardiovascular disease (CVD) (4), and it has been reported that appropriate treatment of T2DM patients can significantly reduce CVD (5). Moreover, CVD remains one of the leading causes of death and disability in patients with T2DM (6–8). In addition to the increased mortality inherent in people with diabetes, mortality nearly doubles when T2DM is associated with CVD manifestations, resulting in a reduction in life expectancy of approximately 12 years (9). The 2019 Diabetes Treatment Guidelines have been updated and mentioned how to prevent and manage CVD (10), including lifestyle (lifestyle changes are key to preventing T2DM and CVD), lipid profiles, blood glucose, and multifactorial approaches. As the prevalence of T2DM increased, the attributable risk of T2DM-induced CVD increased from 5.4% in 1952–1974 to 8.7% in 1975 and 1998, as Fox reported in the Framingham Heart Study (11). In view of the severity of the disease, it is also important to explore the influencing factors of diabetes complicated with CVD. Importantly, one study reported that elevation of HbA1c to or above the target range was the strongest predictor of stroke and acute myocardial infarction (12).

### 1.2 The aim of this study

Some studies have explored the three types of interventions (diet, exercise, and diet plus exercise) and found their effectiveness in disease control and prevention (13). The prognosis for many cases of type 2 diabetes can be adjusted with lifestyle changes, including maintaining a healthy body weight, consuming a healthy diet, staying appropriately physically active, keeping no smoking and drinking alcohol in moderation [13d]. There is also strong evidence that sedentary behavior and unbalanced eating patterns may play an important role in the development of type 2 diabetes as well as CVD (14). Cluster studies on health risk behaviors (HRBs) associated with diabetes are rare, and understanding the cluster of lifestyle risk factors is important because it can be used in developing prevention strategies. Combinations of undesirable lifestyle behaviors have been shown to have a greater risk of disease than the sum of their respective effects, suggesting that risk factors do not act alone, but interact and have synergies [15,15d]. HRBs are interrelated in the population [15d,^16^], and although some studies have suggested that lifestyle management is important for the management of CVD, to our knowledge, few studies have explored the relationships between patterns of multiple HRBs (including intervention) and CVD.

Considering the rising burden of diabetes associated with CVD, a vigilant strategy to reduce modifiable risk factors for diabesity needs further emphasis in primary care settings. The main objective of this study was to explore how interventions co-occur and to investigate the relationship between identified HRB patterns and T2DM with CVD. The results of this study are expected to provide key information for clinical and policy-level decision-making by healthcare providers, healthcare policymakers, and health economic analysts. Therefore, we proposed the following hypotheses: (1) HRBs and intervention management could affect CVD and related indices among the community of China; (2) the correlation between intervention management and CVD and related indices; and (3) there was an effect between HRBs, intervention management, gender, and CVD.

## 2 Methods

### 2.1 Survey design

Since it has been more than 10 years since the last national survey on chronic complications of diabetes, the information on the prevalence rate of chronic complications needs to be updated. The data of the previous epidemiological survey were mainly from large urban hospitals, and few were from the community population, which had a poor representation of the whole country. It is particularly important to grasp the latest data through the implementation of a rigorous epidemiological survey of chronic diabetes complications nationwide, which will further implement the spirit of the CPC Central Committee and The State Council “Opinions on Deepening the Reform of the Medical and Health System” and “China’s Long-Term Plan for the Prevention and Treatment of Chronic Diseases (2017–2025)”. It will also provide a scientific decision-making basis for the government’s prevention and treatment of diabetes and chronic complications. To this end, the Diabetology Branch of the Chinese Medical Association, together with the Chinese Center for Disease Control and Prevention of Chronic Noncommunicable Diseases, and the Bethune Foundation, The China National Diabetic Complications Study (CNDCS) will be conducted in 31 provinces, autonomous regions, and municipalities in 2018 and 2019. In this study, the risk factors for diabetes with CVD and the influence of retrospective intervention measures on CVD and other complications were discussed by extracting data from some of the four cities (17). The current study is designed and reported according to the Strengthening the Reporting of Observational Studies in Epidemiology (STROBE) checklist.

### 2.2 Sampling method

The survey was carried out based on the China Chronic Disease and its Risk Factors Monitoring System, covering 31 provinces (autonomous regions and municipalities directly under the Central Government) (17). Multistage stratified random sampling was used to select the respondents. Detailed sampling methods are shown as follows.

Phase I: A total of 123 out of 298 monitoring sites of chronic diseases and their risk factors in China were selected as the survey sites. According to the provinces (autonomous regions, municipalities directly under the Central Government), the survey points are divided into two levels: district and county. Two districts and two counties were randomly selected from each province. (1) Xizang and Qinghai are difficult to investigate, so one district and one county are selected in these two provinces, and one more county is selected in Sichuan, Henan, and Shandong provinces with large populations. (2) Tianjin, Shanghai, and Beijing consider that there are few county-level units and adjust county-level units to regional sampling units. Finally, (31 × 4 − 1) = 123 monitoring points were selected from 298 monitoring points at the district and county levels as the investigation points.

Phase II: Chronic diseases and their risk factors in China in 2013 were selected from each selected survey point, and four streets (towns) were measured.

Phase III: In 2013, chronic diseases and their risk factors in China were monitored for diabetic patients and basic male patients. The diabetic patients managed by the general health service were stratified by gender and age first and then by gender and age ratio, and 120 cases were randomly selected for investigation.

### 2.3 Sample size estimation

Based on previous research experience, the prevalence of diabetes was 11%, with an accuracy of 15% (*ε*), *α* = 0.05, Z_1-_ *_α_*_/2_ = 1.96. Using the following formula, the minimum sample size was 700 people. Consider a multifactorial design with a different age and region, analysis, and future follow-up needs. This minimum requirement was used for grade sampling in all regions to ensure that the analysis was performed at multiple stratification levels by each community. Therefore, a total of 1,500 people were surveyed.


$$\text{n}=\frac{(1-\text{p}){\text{ Z}}_{1-\text{α}/2}}{{\text{ϵ}}^{2}p}$$

### 2.4 Inclusion criteria

We included participants who had been diagnosed with T2DM and were >18 years old.

### 2.5 Exclusion criteria

Exclusion criteria were as follows: (1) informed consent was not obtained, (2) pregnant women and those with mental illness, (3) failure to submit a questionnaire, (4) those with congenital or acquired immunodeficiency, (5) incomplete medical examination, and (6) no history of mental illness. The flowchart of this study is shown in **Figure S1**.

### 2.6 Investigation contents and methods

#### 2.6.1 Questionnaire survey

The personal questionnaire was conducted by a uniformly trained investigator using a tablet computer in face-to-face form interview and could not be completed by the respondents themselves. The questionnaire included sociodemographic characteristics, HRBs (such as smoking, drinking alcohol, drinking tea and coffee, dietary behaviors, physical activity, sedentary behaviors, SSB intake, sleep duration, and sedentary behavior), family history of disease, personal history of disease, and female birth history, among others. If the above questions are difficult to answer, they should be answered by the family member who is most familiar with the situation.

#### 2.6.2 Exposure

(1) HRBs, including leisure PA, medium work PA, ST, sleep disorders, nuts, fruit juice, alcohol, smoking, sugar-sweetened beverages (SSBs), walking, and vegetables.

1. Leisure PA was measured as “Do you have intensive exercise or recreation that lasts for at least 10 min and causes a significant increase in breathing and heartbeat, such as long-distance running, swimming, playing football, etc.?”

2. Medium work PA was measured as “Do you have moderate intensity activities in your work, farm work and housework activities lasting more than 10 min? (Moderate intensity activities refer to activities such as sawing wood, washing clothes, and cleaning that require moderate physical strength or cause a mild increase in breathing and heartbeat.)”, “How long do you usually spend in your work, farm work and housework?”, and “How long do you usually engage in this intensive exercise or entertainment in a day?”

3. Walking was measured as “Do you walk or bike for at least 10 min when you are out?”

4. Sedentary behaviors were measured as “How much time do you usually sit, lean, or lie down in a day? (Includes time for all static behaviors like sitting and working, studying, reading, watching TV, using a computer, and taking a rest, but not bedtime.)”

5. Sleep disorders were questioned as “Have you had the following sleep problems at least 3 days a week in the past 30 days” [including snoring, choking, or suffocating; difficulty falling asleep (over 30 min); waking up twice or more; waking up early and finding it hard to sleep again; taking sleeping pills (Western medicine or traditional Chinese medicine) for at least 1 day in the past 30 days to help you sleep]? Participants answered yes or no. Then, we summed these answers to a total score, further manipulating other analyses.

6. Nuts were questioned as follows: “Please recall whether you have eaten nuts in the past 12 months and estimate the frequency and amount of various foods.”

7. Fruit juice was questioned as follows: “Please recall whether you have drank fruit juice in the past 12 months and estimate the frequency and amount of various foods.”

8. Alcohol was questioned as “Have you ever had alcohol in the past 12 months?”

9. Smoking was questioned as follows: “Do you smoke now, smoke every day, do not smoke every day, or do not smoke?”

10. SSBs were measured as “Please recall whether you have eaten any of the following foods on a regular basis in the past 12 months and estimate the frequency and amount of each food”. Participants answered as follows: “Whether or not to eat, the frequency of consumption, and the amount of each consumption”. In the analysis of this study, the consumption of each SSB was used.

11. Vegetables were measured as follows: “Please recall whether you have eaten vegetables in the past 12 months and estimate the frequency and amount of various foods.”

(2) Exercise and diet interventions

Participants were asked questions through a questionnaire about exercise and diet interventions. They were asked, “Are you taking steps to control your blood glucose?” If they answered yes, they were asked, “What are the following measures do you take now?” There were three answers: exercise, diet, and drug treatment (including oral antidiabetic drugs and insulin).

Then, these participants were divided into groups treated with diet and exercise alone, diet plus exercise, diet and exercise plus one antidiabetic drug, and neither.

(3) CVH (cardiovascular health)

In 2010, the American Heart Association (AHA) published “Life’s Simple 7” (LS7) to measure CVH and predict future CVD risk. Ideal cardiovascular health, a concept well supported in the literature, is defined by the presence of both ideal health behaviors, which were described previously, including nonsmoking, BMI<25 kg/m^2^, the amount of activity required to achieve the goal, and strive to meet dietary intake in line with current guidelines and favorable health factors (untreated total cholesterol<200 mg/dl, untreated blood pressure<120/80 mm Hg, and fasting blood glucose<100 mg/dl) (18, 19).

#### 2.6.3 Outcomes

Cardiovascular events consisted of five cardiovascular and cerebrovascular events, including angina pectoris, myocardial infarction, cardiac insufficiency, cerebral hemorrhage, and cerebral infarction. Because this is a retrospective study, information related to cardiovascular and cerebrovascular diseases was collected by consulting the patient’s records. Examples include “Have you ever been diagnosed with angina pectoris by a doctor (township/town/street health center and above hospital)?”, “Have you ever been diagnosed with myocardial infarction by a doctor (township/town/street health center and above hospital)?”, “Have you ever been diagnosed with cardiac insufficiency by a doctor (township/town/street health center and above hospital)?”, “Have you ever been diagnosed with cerebral hemorrhage by doctors in medical institutions at the county/district level or above?”, and “Have you ever been diagnosed with a cerebral infarction by a doctor in a medical institution at the county/district level or above?”. The diagnosis of myocardial infarction by a doctor included coronary artery bypass grafting and coronary intervention. Information on all participants’ adverse events was collected by the responsible physician at each visit and reported by the patient in the archival system. The competent doctor of the township/town/street health center and above checked for the above incidents during the participant’s visit, informed the patient, and reported to the local CDC.

### 2.7 Body measurement

Body measurements included height, weight, waist circumference, blood pressure, and pulse. Height measures TGZ height with a maximum scale of 2.0 m and a precision of 0.1 cm; weight measures Billiida (TANITA) HD-390 with a minimum unit of 0.1 kg; waist circumference with a maximum scale of 1.5 m, 1 cm width, and a precision of 0.1 cm; blood pressure and pulse measurements using an Omron HBP1300 electronic sphygmomanometer.

### 2.8 Laboratory testing

Venous blood (5 ml) from each subject was extracted and divided into fluoxalic acid, EDTA, and ordinary test tubes. All direct biochemical measurements were performed using automated chemical analyzers and off-the-shelf reagent kits according to standardized protocols provided by the manufacturer. Venous blood was collected 10 ~ after 12 h, and random urine samples were collected. Detection measures included fasting plasma glucose, glycosylated hemoglobin (HbA1c), blood lipids [serum total cholesterol (TC), serum triglycerides (TGs), serum high-density lipoprotein cholesterol (HDL-C), and serum low-density lipoprotein cholesterol (LDL-C)], liver function [glutamate transaminase (ALT), glutamate transaminase (AST), glutamyl transpeptidase (γ-GGT), total protein, albumin, and globulin], renal function (urea, creatinine, and blood uric acid), and urine albumin/urine creatinine ratio detection, among others. Blood glucose was tested by the local laboratory that passed the survey site; other blood and urine samples were centrifuged and packaged at the investigation site and stored as needed, collected by the national project working group and frozen and transported to the medical inspection institution designated by the National Project Working Group for testing and preservation.

### 2.9 Data collection and management

The data collection of each survey content of each survey point shall be the survey software uniformly compiled and issued by the national project working group. After the end of the daily survey, the tablet network needs to be uploaded to the central server. Each provincial project working group shall regularly check the quality of data input from each survey point and provide timely feedback on any problems. After the provincial data report, the national project working group will clean up the data at the investigation point, finally summarize the national data for analysis, and feedback the cleaned database to the provincial project working group; the provincial project working group will then feedback the data to each investigation point.

### 2.10 Statistical analysis

All the data were analyzed using SPSS 23.0. The demographic data of the participants that fit the normal distribution were represented by means ± standard deviations, and those that did not fit the normal distribution were represented by the median of the range of quartiles (IQR). Multivariate logistic regression was used to explore the correlation between different factors and the risk of diabetes with CVD (20). CVDs were calculated as the total score to determine whether there was CVD, myocardial infarction, cardiac insufficiency, cerebral hemorrhage, or cerebral infarction.

### 2.11 Covariates

Gender, age, education level, residential area, total annual household income, marital status, and ethnicity were included as covariates. The SES variable was created using latent class analysis based on family income level, occupation, education level, and health insurance (each factor had two or four levels) (21).

### 2.12 Sensitivity analysis

In this study, sensitivity analysis was used to test the robustness of the model: (1) The *Z* score of cluster metabolic risk was calculated by adding waist circumference, fasting triglyceride, glycosylated hemoglobin, standardized systolic blood pressure, and reciprocal of HDL-cholesterol to calculate the cluster metabolic risk (zMS) score. The variables are normalized by subtracting the sample mean from the individual mean and dividing the difference by the standard deviation (22). (2) The triglyceride glucose (TyG) index, which includes the measurement of fasting glucose and triglycerides and other outcomes, has been introduced as a reliable substitute marker for insulin resistance (23). Referring to previous studies and the close relationship between insulin resistance and obesity, TyG-related parameters, including TyG and anthropometric indicators of obesity (such as TyG-body mass index [BMI] and TyG-waist circumference [WC]), were superior to TyG alone in predicting insulin resistance [23d]. Therefore, this study also discussed TyG-BMI and TyG-WC (24). VAI: Men: [WC/(39.68 + 1.88 × BMI)] × (TG/1.03) × (1.31/HDL); Women: [WC/(36.58 + 1.89 × BMI)] × (TG/0.81) × (1.52/HDL), where both TG and HDL levels are expressed in mmol/L [23,23d]. CVH was calculated as described in a previous study (18, 19).

## 3 Results

### 3.1 The prevalence of multifactorial intervention

In **Table 1**, elderly age (*χ* ^2^ = 16.6), male sex (*χ* ^2^ = 15.91), lower educational level (*χ* ^2^ = 87.69), lower income (*χ* ^2^ = 59.78), occupation (*χ* ^2^ = 40.32), and lower SES (*χ* ^2^ = 96.02) were correlated with no intervention. Moreover, glycopenia (*χ* ^2^ = 25.05) and a higher level of TyG-WC (*χ* ^2^ = 13.32) were correlated with no intervention.

**Table 1:** The prevalence of multifactorial intervention.

	Total	All none	Exercise + no diet	No exercise + diet	All have	*χ* ^2^/*F* value
Age						16.60**
≤59.10	990	150 (15.2)	36 (3.6)	435 (43.9)	369 (37.3)	
>59.10	775	120 (15.5)	21 (2.7)	277 (35.7)	357 (46.1)	
Gender						15.91**
Male	873	140 (16.0)	33 (3.8)	312 (35.7)	388 (44.4)	
Female	892	130 (14.6)	24 (2.7)	400 (44.8)	338 (37.9)	
Ethnicity						4.80
Han	1,745	269 (15.4)	55 (3.2)	705 (40.4)	716 (41.0)	
No-Han	20	1 (5.0)	2 (10.0)	7 (35.0)	10 (50.0)	
Marital status						4.55
No	30	8 (26.7)	0 (0.0)	13 (43.3)	9 (30.0)	
Yes	1,735	262 (15.1)	57 (3.3)	699 (40.3)	717 (41.3.)	
Education level						87.69**
Below primary school level	800	139 (17.4)	16 (2.0)	381 (47.6)	264 (33.0)	
Primary school	262	38 (14.5)	14 (5.3)	108 (41.2)	102 (38.9)	
Junior high school	472	67 (14.2)	21 (4.4)	170 (36.0)	214 (45.3)	
Senior high school	231	26 (11.3)	6 (2.6)	53 (22.9)	146 (63.2)	
Total annual household income (yuan)						59.78**
≤18,000	354	76 (21.5)	10 (2.8)	156 (44.1)	112 (31.6)	
18,000–40,000	749	125 (16.7)	27 (3.6)	320 (42.7)	277 (37.0)	
40,000–70,000	346	26 (7.5)	10 (2.9)	137 (39.6)	173 (50.0)	
>70,000	316	43 (13.6)	10 (3.2)	99 (31.3)	164 (51.9)	
Occupation level						40.32**
No	653	77 (11.8)	20 (3.1)	225 (34.5)	331 (50.7)	
Yes	1,112	193 (17.4)	37 (3.3)	487 (43.8)	395 (35.5)	
Insurance level						2.05
No	53	6 (11.3)	1 (1.9)	26 (49.1)	20 (37.7)	
Yes	1,712	264 (15.4)	56 (3.3)	686 (40.1)	706 (41.2)	
Social economic status						96.02**
Low	835	158 (18.9)	26 (3.1)	394 (47.2)	257 (30.8)	
Medium	461	63 (13.7)	13 (2.8)	186 (40.3)	199 (43.2)	
High	469	49 (10.4)	18 (3.8)	132 (28.1)	270 (57.6)	
FBG						25.05**
<6.1	598	47 (25.5)	3 (1.6)	61 (33.2)	73 (39.7)	
6.1–6.99	5,228	27 (11.8)	3 (1.3)	91 (39.9)	107 (46.9)	
≥7.0	1,350	196 (14.5)	51 (3.8)	558 (41.3)	545 (40.4)	
VSI						6.08
High	580	99 (17.1)	19 (3.3)	241 (41.6)	221 (38.1)	
Medium	598	88 (14.7)	19 (3.2)	247 (41.3)	244 (40.8)	
Low	580	82 (14.1)	19 (3.3)	220 (37.9)	259 (44.7)	
TyG						11.3
High	580	90 (15.5)	26 (4.5)	244 (42.1)	220 (37.9)	
Medium	598	98 (16.4)	13 (2.2)	245 (41.0)	242 (40.5)	
Low	580	81 (14.0)	18 (3.1)	219 (37.8)	262 (45.2)	
TyG/HDL						6.01
High	580	99 (17.1)	22 (3.8)	237 (40.9)	222 (38.3)	
Medium	598	85 (14.2)	16 (2.7)	249 (41.6)	248 (41.5)	
Low	580	85 (14.7)	19 (3.3)	222 (38.3)	254 (43.8)	
TyG-BMI						9.47
High	580	102 (17.6)	24 (4.1)	225 (38.8)	229 (39.5)	
Medium	598	85 (14.2)	17 (2.8)	259 (43.3)	237 (39.6)	
Low	580	82 (14.1)	16 (2.8)	224 (38.6)	258 (44.5)	
TyG-WC						13.22*
High	580	103 (17.8)	23 (4.0)	221 (38.1)	233 (40.2)	
Medium	598	83 (13.9)	20 (3.3)	266 (44.5)	229 (38.3)	
Low	580	83 (14.3)	14 (2.4)	221 (38.1)	262 (45.2)	
zMS						8.46
High	580	103 (17.8)	23 (4.0)	233 (40.2)	221 (38.1)	
Medium	598	84 (14.0)	20 (3.3)	247 (41.3)	247 (41.3)	
Low	580	82 (14.1)	14 (2.4)	228 (39.3)	256 (44.1)	

*, P<0.05, **, P<0.01.

### 3.2 The prevalence of CVD

In **Table 2**, elderly age (*χ* ^2^ = 22.70), no occupation (*χ* ^2^ = 20.97), medium and high SES (*χ* ^2^ = 19.92), higher TyG-WC (*χ* ^2^ = 6.60), and higher zMS (*χ* ^2^ = 7.59) were correlated with high CVD when compared with lower levels. Others were not significant.

**Table 2:** The prevalence of CVD.

	Total	None	≥1	*χ* ^2^ value
Age				22.70**
≤59.10	990	976 (98.6)	14 (1.4)	
>59.10	775	733 (94.6)	42 (5.4)	
Gender				1.01
Male	873	849 (97.3)	24 (2.7)	
Female	892	860 (96.4)	32 (3.6)	
Ethnicity				0.66
Han	1,745	1,689 (96.8)	56 (3.2)	
No-Han	20	20 (100)	0 (0.0)	
Marital status				1.0
No	30	30 (100)	0 (0.0)	
Yes	1,735	1,679 (96.8)	56 (3.2)	
Education level				2.48
Below primary school level	800	774 (96.8)	26 (3.3)	
Primary school	262	251 (95.8)	11 (4.2)	
Junior high school	472	457 (96.8)	15 (3.2)	
Senior high school	231	227 (98.3)	4 (1.7)	
Total annual household income (yuan)				3.34
≤18,000	354	341 (96.3)	13 (3.7)	
18,000–40,000	749	724 (96.7)	25 (3.3)	
40,000–70,000	346	333 (96.2)	13 (3.8)	
>70,000	316	311 (98.4)	5 (1.6)	
Occupation level				20.97**
No	653	616 (94.3)	37 (5.7)	
Yes	1,112	1,093 (98.3)	19 (1.7)	
Insurance level				0.06
No	53	51 (96.2)	2 (3.8)	
Yes	1,712	1,658 (96.8)	54 (3.2)	
Social economic status				19.92**
Low	835	819 (98.1)	16 (1.9)	
Medium	461	432 (93.7)	29 (6.3)	
High	469	458 (97.7)	11 (2.3)	
FBG				5.43
<6.1	184	179 (97.3)	5 (2.7)	
6.1–6.99	228	215 (94.3)	13 (5.7)	
≥7.0	1,350	1,312 (97.2)	38 (2.8)	
VSI				3.80
High	580	560 (96.6)	20 (3.4)	
Medium	598	574 (96.0)	24 (4.0)	
Low	580	568 (97.9)	12 (2.1)	
TyG				2.46
High	580	562 (96.9)	18 (3.1)	
Medium	598	574 (96.0)	24 (4.0)	
Low	580	566 (97.6)	14 (2.4)	
TyG/HDL				1.72
High	580	560 (96.6)	20 (3.4)	
Medium	598	576 (96.3)	22 (3.7)	
Low	580	566 (97.6)	14 (2.4)	
TyG-BMI				3.73
High	580	555 (95.7)	25 (4.3)	
Medium	598	581 (97.2)	17 (2.8)	
Low	580	566 (97.6)	14 (2.4)	
TyG-WC				6.60*
High	580	555 (95.7)	25 (4.3)	
Medium	598	577 (96.5)	21 (3.5)	
Low	580	570 (98.3)	10 (1.7)	
zMS				7.59*
High	580	563 (97.1)	17 (2.9)	
Medium	598	570 (95.3)	28 (4.7)	
Low	580	569 (98.1)	11 (1.9)	
Behavioral and dietary interventions				0.37
All none	270	263 (97.4)	7 (2.6)	
Exercise + no diet	57	55 (95.6)	2 (3.5)	
No exercise + diet	712	689 (96.8)	23 (3.2)	
All have	726	702 (96.7)	24 (3.3)	

*, P<0.05, **, P<0.01.

### 3.3 Linear regression analysis between health risk behaviors and zMS (sensitivity analysis)

In **Table 3**, there was a dose−response relationship between HRB cooccurrence and zMS (*β* = 0.04, *p*< 0.01). After controlling for covariates, this relationship was not significant (*β* = 0.02, *p* = 0.103). There was also a dose−response relationship between the clustering of HRBs and zMS (*β* = 0.03, *p*< 0.01); after controlling for covariates, this relationship was not significant (*β* = 0.01, *p* = 0.224). There was no dose−response relationship between MVPA and zMS (*β* = −0.011, *p* = 0.06); after controlling for covariates, this relationship was not significant (*β* = −0.01, *p* = 0.121). There was no dose−response relationship between SB and zMS (*β* = 0.02, P=365); after controlling for covariates, this relationship was not significant (*β* = 0.03, *p* = 0.168). There was a dose−response relationship between walking and zMS (*β* = −0.844, *p* = 0.013); after controlling for covariates, this relationship was not significant (*β* = 0.01, *p* = 0.240). There was a borderline dose−response relationship between high-intensity PA and zMS (*β* = −0.261, *p* = 0.063); after controlling for covariates, this relationship was not significant (*β* = −0.231, *p* = 0.104).

**Table 3:** The multilevel linear regression between health risk behaviors (numbers) and zMS.

HRBs co-occurrence	*R* ^2^	*β*	*t*	*p*	*F*	LLCI	ULCI
Model 1	0.01	0.04	3.89	**<0.01**	15.13	0.02	0.03
Model 2	0.18	0.02	1.63	0.103	55.10	−0.003	0.034
HRBs clustering							
Model 1	0.01	0.03	4.135	**<0.01**	17.101	0.016	0.044
Model 2	0.293	0.01	1.216	0.224	103.625	−0.01	0.02
MVPA							
Model 1	0.002	−0.011	−1.88	0.06	2.54	−0.022	0.000
Model 2	0.038	−0.01	−1.553	0.121	9.767	−0.02	0.002
SB							
Model 1	0.000	0.02	0.91	0.365	0.821	−0.023	0.064
Model 2	0.222	0.03	1.38	0.168	12.963	−0.013	0.073
Walking							
Model 1	0.003	−0.844	−2.477	**0.013**	6.14	−1.513	−0.176
Model 2	0.032	0.01	1.175	0.240	8.31	−0.003	0.013
High intensity PA							
Model 1	0.002	−0.261	−1.858	0.063	3.454	−0.536	0.014
Model 2	0.014	−0.231	−1.628	0.104	3.462	−0.509	0.047

Model 1: crude model; Model 2: controlled for educational level, marital status, total annual household income, ethnicity, gender, and age. Bold values mean significant.

### 3.4 Linear regression analysis between multifactorial intervention and CVH (sensitivity analysis)

In **Table 4**, there was a dose−response relationship between multifactorial intervention and CVH (*β* = 0.294, *p*< 0.01); after controlling for covariates, this relationship was not significant (*β* = 0.253, *p*< 0.01). In **Table 5**, there was a dose−response relationship between multifactorial intervention and CVH (*β* = 0.261, *p*< 0.01); after controlling for covariates, this relationship was not significant (*β* = 0.219, *p*< 0.01).

**Table 4:** The multilevel linear regression between CVH and multifactorial intervention (behavioral, diet, and drug treatment).

Multifactorial intervention	CVH						
	*R* ^2^	*β*	*t*	*p*	*F*	LLCI	ULCI
Model 1	0.015	0.294	5.206	**<0.01**	27.103	0.183	0.405
Model 2	0.085	0.253	4.525	**<0.01**	23.21	0.143	0.363

Model 1: crude model; Model 2: controlled for educational level, marital status, total annual household income, ethnicity, gender, and age. Bold values mean significant.

**Table 5:** The multilevel linear regression between CVH and multifactorial intervention (behavioral, diet, drug treatment, and weight management).

Multifactorial intervention	CVH						
	*R* ^2^	*β*	*t*	*p*	*F*	LLCI	ULCI
Model 1	0.017	0.261	5.450	**<0.01**	29.704	0.167	0.355
Model 2	0.086	0.219	4.611	**<0.01**	23.332	0.126	0.312

Model 1: crude model; Model 2: controlled for educational level, marital status, total annual household income, ethnicity, gender, and age. Bold values mean significant.

### 3.5 Moderation analysis between health risk behaviors and zMS (sensitivity analysis)

In **Tables 6** and **7**, age moderates the association between HRB cooccurrence (coeff = −0.31), clustering of HRBs (coeff = −0.58) and zMS.

**Table 6:** The moderation analysis between zMS, HRB co-occurrence, and age.

Variables	zMS					
	coeff	se	*t*	*p*	LLCI	ULCI
HRB co-occurrence	0.66	0.17	3.96	**<0.01**	0.33	0.98
Age	3.47	1.32	2.63	**<0.01**	0.88	6.07
Int_1	−0.31	0.11	−2.85	**<0.01**	−0.53	−0.10

Int 1: HRB co-occurrence × age. Bold values mean significant.

**Table 7:** The moderation analysis between zMS, HRB clustering, and age.

Variables	zMS					
	coeff	se	*t*	*p*	LLCI	ULCI
Clustering of HRB	1.13	0.24	4.75	**<0.01**	0.66	1.59
Age	0.62	0.27	2.29	**<0.05**	0.1	1.16
Int_1	−0.58	0.16	−3.63	**<0.01**	−0.90	−0.27

Int 1: HRB clustering × age. Bold values mean significant.

### 3.6 The mediation moderation analysis between health risk behaviors and CVD

In **Table 8** and **Table 9**, the clustering of HRBs was correlated with TyG (*β* = 0.20) but was not correlated with CVD (*β* = 0.01). TyG was correlated with CVD, and there was an association between sex, TyG, and CVD (*β* = −0.10). This finding indicates that gender plays a moderating role in TyG-induced CVD.

**Table 8:** Model characteristics for the conditional process analysis.

Variables	TyG			CVD		
	*β*	t value	*p* value	*β*	*t* value	*p* value
HRB	0.20	2.33	**<0.05**	0.01	0.18	>0.05
Gender	0.16	1.67	>0.05	0.98	2.92	**<0.01**
HRB*Gender	−0.13	−1.95	**0.0515**	0.01	0.19	>0.05
TyG				0.17	3.28	**<0.01**
Gender*TyG				−0.10	−2.97	**<0.01**
*R* ^2^		0.004			0.01	
*F*		2.44			2.56	

Mediation variable: TyG; moderated variable: gender; independent variable: HRB; dependent variable: CVD, Uncontrolled. Bold values mean significant.

**Table 9:** Bootstrapped conditional direct and indirect effects.

			HRB clustering		
Direct effect			Effect	SE	(LL,UL)
	Predictor	SERF			
	Moderator (gender)	Male	0.0202	0.0209	−0.0208, 0.0613
		Female	0.0293	0.0446	−0.0582, 0.1169
Indirect effect			Effect	SE	(LL,UL)
	Predictor	HRB			
	Mediator (TyG)	Low	0.0044	0.0024	0.0004, 0.010
		High	0.0024	0.0028	−0.0009, 0.0123

### 3.7 Moderation analysis between health risk behaviors, TyG-BMI, and CVD

In **Tables 10** and **11**, age moderates the association between HRB clustering of HRB and TyG-BMI (coeff = −6.50) and TyG-WC (coeff = −26.03).

**Table 10:** The moderation analysis between TyG-BMI, HRB clustering, and age.

Variables	TyG-BMI					
	coeff	se	*t*	*p*	LLCI	ULCI
Clustering of HRBs	11.16	3.78	2.96	**<0.01**	3.75	18.56
Age	−0.18	4.23	−0.04	>0.05	−8.48	8.12
Int_1	−6.50	2.48	−2.62	**<0.01**	−11.36	−1.63

Int 1: Clustering of HRBs × age. Bold values mean significant.

**Table 11:** The moderation analysis between TyG-WC, HRB clustering, and age.

Variables	TyG-WC					
	coeff	Se	*t*	*p*	LLCI	ULCI
Clustering of HRBs	44.36	11.06	4.01	**<0.01**	22.67	66.06
Age	18.58	12.39	1.50	>0.05	−5.73	42.89
Int_1	−26.03	7.27	−3.58	**<0.01**	−40.28	−11.77

Int 1: Clustering of HRBs × age. Bold values mean significant.

Moderation analyses were performed with educational level, marital status, total annual household income, ethnicity, and gender as the control variables. The results are presented in **Table 11**. First, HRBs did not predict the severity of CVD (*β* = 0.42), TyG-BMI was also not associated with CVD, and HRBs×age significantly correlated with the severity of CVD. There was a three-way interaction effect between HRBs, TyG-BMI, and age. After controlling for covariates, these results were also significant.

### 3.8 Logistic regression analysis between behavioral and dietary interventions and CVD

In **Tables 12**–**14**, we show the correlation between intervention management (behavioral and dietary interventions, including diet and exercise) and CVD. Interestingly, compared with both behaviors, the participants with neither behavior showed meaningless results, while the participants with exercise and no dietary control showed meaningful results, which were more than three times larger. Moreover, we also stratified by sex, and female participants were more sensitive to CVD.

**Table 12:** The correlation between intervene and cardiovascular diseases.

Variables	Model 1		Model 2	
	None	Yes	None	Yes
No exercise + no diet	1.0	1.10 (0.54,2.25)	1.0	1.11 (0.53,2.31)
Exercise + no diet	1.0	3.05 (1.20,7.71)*	1.0	3.65 (1.41,9.50)**
No exercise + diet	1.0	1.38 (0.83,2.30)	1.0	1.50 (0.88,2.54)
Exercise + diet	1.0	1.0	1.0	1.0

Model 1: crude model; model 2: controlling for age, educational level, marital status, total annual household income, ethnicity, and gender. *, P<0.05, **, P<0.01. Bold values mean significant.

**Table 13:** The correlation between intervene and CVD (male).

Variables	Model 1		Model 2	
	None	Yes	None	Yes
No exercise + no diet	1.0	0.55 (0.16,1.91)	1.0	0.57 (0.16,2.04)
Exercise + no diet	1.0	0.78 (0.10,6.07)	1.0	1.17 (0.15,9.44)
No exercise + diet	1.0	1.17 (0.56,2.46)	1.0	1.39 (0.63,3.05)
Exercise + diet	1.0	1.0	1.0	1.0

Model 1: crude model; model 2: controlling for age, educational level, marital status, total annual household income, ethnicity, and gender.

**Table 14:** The correlation between intervene and CVD (female).

Variables	Model 1		Model 2	
	None	Yes	None	Yes
No exercise + no diet	1.0	1.78 (0.71,4.46)	1.0	1.82 (0.71,4.64)
Exercise + no diet	1.0	7.15 (2.28,22.38)	1.0	7.11 (2.20,22.94)**
No exercise + diet	1.0	1.58 (0.77,3.24)	1.0	1.71 (0.82,3.58)
Exercise + diet	1.0	1.0	1.0	1.0

Model 1: crude model; model 2: controlling for age, educational level, marital status, total annual household income, ethnicity, and gender. **, P<0.01.

### 3.9 Logistic regression analysis between behavioral and dietary interventions (two types) and CVD

In **Tables 15**–**17**, we show the correlation between intervention management (behavioral and dietary interventions, including diet and exercise) and CVD. Interestingly, compared with both behaviors, the participants with neither behavior showed meaningless results, while the participants with exercise and no dietary control showed significant results, which were more than three times larger. Moreover, we also stratified by sex, and female participants were more sensitive to CVD.

**Table 15:** The correlation between intervene and CVD.

Variables	Model 1		Model 2	
	None	Yes	None	Yes
Exercise and diet				
No exercise + no diet	1.0	0.73 (0.50,1.05)	1.0	0.72 (0.49,1.06)
Exercise + no diet	1.0	1.46 (0.80,2.68)	1.0	1.78 (0.94,3.37)
No exercise + diet	1.0	0.91 (0.70,1.18)	1.0	1.0 (0.76,1.31)
Exercise + diet	1.0	1.0	1.0	1.0

Model 1: crude model; model 2: controlling for age, educational level, marital status, total annual household income, ethnicity, and gender

**Table 16:** The correlation between intervene and CVD (male).

Variables	Model 1		Model 2	
	None	Yes	None	Yes
Exercise and diet				
No exercise + no diet	1.0	0.66 (0.39,1.10)	1.0	0.70 (0.41,1.21)
Exercise + no diet	1.0	0.78 (0.31,1.95)	1.0	1.10 (0.42,2.85)
No exercise + diet	1.0	0.82 (0.57,1.19)	1.0	0.99 (0.66,1.49)
Exercise + diet	1.0	1.0	1.0	1.0

Model 1: crude model; model 2: controlling for age, educational level, marital status, total annual household income, ethnicity, and gender.

**Table 17:** The correlation between intervene and CVD (female).

Variables	Model 1		Model 2	
	None	Yes	None	Yes
Exercise and diet				
No exercise + no diet	1.0	0.82 (0.49,1.40)	1.0	0.75 (0.43,1.30)
Exercise + no diet	1.0	2.89 (1.23,6.79)*	1.0	3.02 (1.22,7.48)*
No exercise + diet	1.0	1.01 (0.70,1.45)	1.0	1.01 (0.69,1.49)
Exercise + diet	1.0	1.0	1.0	1.0

Model 1: crude model; model 2: controlling for age, educational level, marital status, total annual household income, ethnicity, and gender. **, P<0.05.

Moderation analyses were performed with age, educational level, marital status, total annual household income, and ethnicity as the control variables. There was an association between intervention management, zMS, TyG-WC, TyG-BMI, sex, and CVD. The results are presented in **Tables 18**–**20** and **Table S1**. VSI, TyG, and WHtR showed no significant results.

**Table 18:** The moderation analysis between HRBs, zMS, age, and CVD.

Variables	CVD					
	coeff	se	*t*	*p*	LLCI	ULCI
Intervene	−0.21	0.10	−2.20	**<0.05**	−0.41	−0.02
zMS	−0.28	0.15	−1.85	>0.05	−0.58	0.02
Gender	−0.43	0.21	−2.08	**<0.05**	−0.83	−0.02
Int_1	0.10	0.05	2.07	**<0.05**	0.005	0.26
Int_2	0.14	0.06	2.10	**<0.05**	0.009	0.26
Int_3	0.20	0.10	2.08	**<0.05**	0.01	0.40
Int_4	−0.06	0.03	−2.12	**<0.05**	−0.12	−0.21

Int 1: Intervene × zMS; Int 2: Intervene × gender; Int 3: zMS × gender; Int 4: Intervene × zMS × gender. Controlled for age, educational level, marital status, total annual household income, and ethnicity.

**Table 19:** The moderation analysis between HRBs, TyG-WC, age, and CVD.

Variables	CVD					
	coeff	se	*t*	*p*	LLCI	ULCI
Intervene	−0.19	0.10	−2.02	**<0.05**	−0.38	−0.006
TyG-WC	−0.30	0.15	−2.04	**<0.05**	−0.60	−0.01
Gender	−0.49	0.21	−2.40	**<0.05**	−0.90	−0.09
Int_1	0.09	0.05	1.92	>0.05	−0.002	0.18
Int_2	0.13	0.06	1.97	**<0.05**	0.0003	0.25
Int_3	0.24	0.03	2.45	**<0.05**	0.047	0.43
Int_4	−0.06	0.026	−2.01	**<0.05**	−0.12	−0.0016

Int 1: Intervene × TyG-WC; Int 2: Intervene × gender; Int 3: TyG-WC × gender; Int 4: Intervene × TyG-WC × gender. Controlled for age, educational level, marital status, total annual household income, and ethnicity.

**Table 20:** The moderation analysis between HRBs, TyG-BMI, age, and CVD.

Variables	CVD					
	coeff	se	*t*	*p*	LLCI	ULCI
Intervene	−0.22	0.10	−2.24	**<0.05**	−0.42	−0.027
TyG-BMI	−0.32	0.15	−2.15	**<0.05**	−0.62	−0.028
Gender	−0.54	0.30	−2.66	**<0.01**	−0.94	−0.14
Int_1	0.10	0.046	2.18	**<0.05**	0.01	0.19
Int_2	0.16	0.06	2.50	**<0.05**	0.03	0.28
Int_3	0.27	0.10	2.78	**<0.01**	0.08	0.46
Int_4	−0.08	0.03	−2.59	**<0.01**	−0.14	−0.019

Int 1: Intervene × TyG-BMI; Int 2: Intervene × gender; Int 3: TyG-BMI × gender; Int 4: Intervene × TyG-BMI × gender. Controlled for age, educational level, marital status, total annual household income, and ethnicity.

### 3.10 Moderation analysis between behavioral and dietary interventions (three types) and CVD (sensitivity analysis)

Moderation analyses were performed with age, educational level, marital status, total annual household income, and ethnicity as the control variables. There was an association between intervention management (behavioral and dietary interventions including diet, exercise, and drug treatment), zMS, TyG-BMI, gender, and CVD. The results are presented in **Tables S2** and **S3**. VSI, TyG, TyG-WC, and WHtR showed no significant results. The mediating moderation analysis figure is shown in **Table S2**.

### 3.11 The moderate mediation analysis between behavioral and dietary interventions (three types), CVH, and CVD (sensitivity analysis)

Behavioral and dietary interventions included diet, exercise, and drug treatment; CVH included BMI, TC, moderate PA, healthy diet score (whole grain, poultry, vegetable, SSBs, and milk product), SBP, and FBG. In **Tables S4** and **S5**, intervention management was not correlated with CVH (*β* = −0.038) but was correlated with CVD (*β* = 0.013). CVH was correlated with CVD, and there was an association between sex, CVH, and CVD (*β* = −0.10). This finding indicates that sex plays a regulatory role in CVH-induced CVD.

In **Tables S6** and **S7**, the clustering of HRB was correlated with CVH (*β* = 0.20) but was correlated with CVD (*β* = 0.01). TyG was correlated with CVD, and there was an association between sex, HbA1c, CVH, and CVD (*β* = −0.10). This finding indicates that sex plays a moderating role in TyG-induced CVD. The mediating moderation analysis figure is shown in **Table S3**.

### 3.12 Logistic regression analysis between behavioral and dietary interventions and metabolic index

In **Table S8**, we show the correlation between the metabolic index and behavioral and dietary interventions. These metabolic indices were all correlated with CVD. Compared with both behaviors, the participants with neither behavior showed meaningless results, and the participants with exercise and no dietary control also showed significant results.

## 4 Discussion

### 4.1 Principal findings

First, the overall prevalence of CVD was 19.9% among diabetic participants, which was similar to a previous study (17, 25) but lower than other studies (26, 27). Second, we also found that clinical intervention (including exercise, diet, and drug treatment) was negatively correlated with the occurrence and development of CVD, clustering of HRBs may contribute to clinical intervention, and there were correlations between clinical intervention, metabolic index, and CVD; this is supplemented by previous studies that demonstrated methods of preventing diabetes and complication risk by modeling risk factors. Third, effective interventions help to reduce the occurrence and development of CVD (whether it is two or three interventions), and the complementary effects of risk factors will increase the incidence of CVD. There is a correlation between intervention measures and various metabolic indicators and CVD, and the interaction between HRB and various metabolic indicators also has an impact on CVD. Through a series of sensitivity analyses, this study further verified that reasonable and effective interventions focusing on diet control and exercise can also help reduce the occurrence of CVD. Finally, in the moderation analysis, sex and metabolic index moderated the correlation between clinical intervention and CVD. Preventive interventions for T2DM can provide a theoretical basis for risk score assessment (including this study) to predict diabetes (28). Our results suggest that further strengthening of multilevel interventions can significantly reduce the incidence of cardiovascular events in T2DM patients compared with no control measures. Moreover, we verified our robustness according to the sensitivity analysis results.

### 4.2 Comparison with other studies

Evidence suggests that T2DM is accompanied by an increase in a series of metabolic indices, such as lipid profiles, BMI, and WC, which are also correlated with the occurrence and development of CVD. In our study, we constructed a retrospective intervention model to measure the correlation between these metabolic indices and CVD (29). Studies have also indicated that the most important pathophysiological mechanisms of diabetes with cardiovascular complications are insulin resistance (IR) and hyperglycemia (30); some metabolic indices (zMS, TyG, TyG-BMI, and TyG-WC) in this study can reflect the influence of these two levels. IR is closely associated with obesity, which is commonly found in people with T2DM. Lipid profiles and inflammatory mediators, both present in obese patients, increase the production of reactive oxygen species and systemic inflammation (30). According to the possible mechanism, biomarker inflammatory responses were involved, including high-sensitivity C-reactive protein (31, 32) and oxidative stress (33).

First, the progression of hyperglycemia, hyperlipidemia, IR, and hyperinsulinemia seems to initiate and continue to promote the development of metabolic disease and CVD. Diabetes can cause myocardial ischemia through vascular diseases (microvascular puraemia and macrovascular puraemia) and can directly have deleterious effects on the myocardium (cardiomyocytes and stroma) (30, 34). The interventions mentioned in this study also confirmed that the association with CVD could be measured in combination with control measures, including exercise, diet and medication, and metabolic markers. Lifestyle interventions that reduce body weight and increase physical activity produce many metabolic benefits, especially for T2DM patients with CVD, and there is an association between the amount of weight lost in T2DM subjects and the incidence of CVD (35), which further provides a theoretical basis for our study.

Furthermore, we also explored the correlation between the metabolic index and clinical intervention. In the Ueki study, they found that mean HDL cholesterol concentrations were significantly higher in the intensive treatment group than in the conventional treatment group, possibly due to increased physical activity in the intensive treatment group. These indicators are discussed because studies have linked CVD to blood lipids and smoking (36). In addition, our study added two to four clinical interventions to further describe the impact of clinical interventions on CVD; therefore, the prevalence of T2DM and its chronic complications, particularly CVD, in society seems to be reduced by continuing to control blood lipids and taking preventive measures to halt the growth rate of metabolic diseases. There are some empirical studies that support our findings (37), and they found that unhealthy lifestyles were common, especially among older participants and those without more than a college education. In addition, an unhealthy lifestyle is associated with poor control of fasting blood glucose, lipids, and blood pressure. Interventions to modify risky lifestyle behaviors are needed, and this study is designed to provide theoretical support for reducing the impact of T2DM diabetes and CVD in developing countries by asking retrospectively about interventions (25).

Third, it is very important to consider sex differences in CVD and associated severity in diabetes. Yamagishi recently summarized the results of multifactorial clinical studies and confirmed in a meta-analysis of 820,000 people that the risk ratio of death from CVD in people with diabetes compared with nondiabetic individuals was 2.32, even after adjusting for statistical measures of age, sex, smoking status, and BMI (38, 39). This and other studies report that women with diabetes have a higher relative risk of cardiovascular death than men. The design of preclinical studies strongly requires recognition of the importance of gender-based differences in CVD because of the recent increasing emphasis in preclinical studies of CVD pathology (40) on the inclusion of both male and female animals and their primary cells. Overall, the consideration of anatomical and sex differences in the treatment of diabetic CVD offers great promise for revealing fundamental new insights into the pathogenesis of CVD and developing new personalized treatment strategies.

Finally, similar to T2DM, there are also some similar risk factors related to the occurrence of CVD. For example, age is a well-known risk independent factor for CVD, and the prevalence of CVD in elderly patients is higher than that in young patients (25). Other factors, such as obesity, are considered independent risk factors for CVD, and studies have shown that overweight and obesity are prevalent in people with T2DM and are at high risk for CVD (25). BMI and waist circumference are associated with major cardiometabolic risk factors, including high blood pressure and elevated levels of LDL cholesterol. Therefore, this study derived a series of complex indicators related to these indicators, such as TyG-BMI, TyG-WC, and zMS. In addition, higher BMI was associated with higher CVD in our study. In our study, we found that higher socioeconomic status is associated with a higher prevalence of CVD, possibly because of the low social and economic status of the country’s high CVD because of medical and health conditions. These high levels of health determinants among fair decision factors, including social and community environment (i.e., food), social environment, and social psychological factors, ultimately affect housing, food insecurity, and fiscal pressures or transportation. To define the social risk, thus affecting the occurrence of CVD (41). The high level of CVD in countries with high economic and living status is due to lifestyle reasons (42).

The results of this study indicate that patients with T2DM have a higher incidence of CVD in the study population. Considering the complexity of the disease, it is particularly important to explore its related influencing factors. Therefore, preventing disease and reducing T2DM-related complications requires a combination of interventions including regular exercise, diet, and medication, as well as regular measurement and control of blood pressure, cholesterol, and blood sugar. Because CVD in diabetic patients is largely preventable, management and treatment can continue even when complications occur, people with diabetes need comprehensive training on disease and prevention methods, and early and timely diagnosis can effectively control and treat complications (43).

### 4.3 Limitations and strengths

Several limitations of this study must be noted. The assessment of clinical interventions and metabolic indices is by self-reported measures, which are subject to imprecision, bias, and inaccuracy. As a cross-sectional study, it is difficult to observe the causal association between metabolic index, health risk factors, and CVD. Although this study explored the effectiveness of the intervention method, its generalization is limited because it is a retrospective study without follow-up intervention measures for patients. Although this series of surveys covers most of the cities in the country, this study only extracts the results of four of them, it is not clear how representative the sample is, and follow-up surveys will continue to be conducted in samples from different regions and cultural backgrounds across the country.

The strengths include the fact that we have reported health risk factors and diabetes and its complications, such as CVD; this study verified clinical intervention effectiveness to explore the correlation between metabolic indices and CVD, which gives us a good theoretical basis. Other strengths of this study are that the data came from a multifactorial and multilevel design and the study involved a large number of participants (17).

## 5 Conclusions

The results of this study revealed that there is a high prevalence rate of CVD in patients with T2DM in Anhui Province. Our findings suggest the potential benefits of scaling up multifactorial and multifaceted interventions to prevent CVD in patients with T2DM. Therefore, appropriate strategies can improve this situation by establishing a hierarchical system of diagnosis and treatment, tracking and monitoring it at all levels, and providing feedback to hospitals, which can provide effective numerical data for reducing financial burden and economic pressure in the future.

## Data availability statement

The raw data supporting the conclusions of this article will be made available by the authors, without undue reservation.

## Ethics statement

The studies involving human participants were reviewed and approved by the Ethics Committee of Anhui Medical University. The patients/participants provided their written informed consent to participate in this study.

## Author contributions

QZ constructed the study design. FD and HH recruited the participants. YZ, FD, and TJ were involved in the statistical analysis. QZ was responsible for critical revision of the manuscript. YZ, TJ, and CL edited and revised the manuscript. YZ and CL prepared and drafted the manuscript. The work presented here has not been published previously and is not being considered for publication elsewhere. All authors contributed to the article and approved the submitted version.
