# Supplementary material for: The management correlation between metabolic index, cardiovascular health, and diabetes combined with cardiovascular disease
Source: Front Endocrinol (Lausanne). 2023 Jan 27;13:1036146. doi: 10.3389/fendo.2022.1036146 (PMC9911412; doi:10.3389/fendo.2022.1036146)
Supplement: Supplementary file 1 [file DataSheet_1.doc]

| Table S1 The moderation analysis between HRBs, TG/HDL, gender and CVD | | | | | | |
| --- | --- | --- | --- | --- | --- | --- |
| Variables | CVD | | | | | |
|  | coeff | se | t | P | LLCI | ULCI |
| Intervene | -0.20 | 0.10 | -2.05 | **＜0.05** | -0.40 | -0.0088 |
| TG/HDL | -0.30 | 0.15 | -2.02 | **＜0.05** | -0.59 | -0.0088 |
| Gender | -0.47 | 0.21 | -2.27 | **＜0.01** | -0.87 | -0.06 |
| Int_1 | 0.09 | 0.05 | 1.94 | ＞0.05 | -0.001 | 0.18 |
| Int_2 | 0.13 | 0.06 | 2.07 | **＜0.05** | 0.07 | 0.26 |
| Int_3 | 0.23 | 0.097 | 2.32 | **＜0.05** | 0.06 | 0.42 |
| Int_4 | -0.06 | 0.03 | -2.08 | **＜0.05** | -0.12 | -0.0037 |
| Int 1: Intervene × TG/HDL; Int 2: Intervene × gender; Int 3: TG/HDL × gender; Int 4: Intervene × TG/HDL × gender. Controlled for age, educational level, marital status, total annual household income and ethnic. | | | | | | |

| Table S2 The moderation analysis between HRBs, TyG-BMI, age and CVD | | | | | | |
| --- | --- | --- | --- | --- | --- | --- |
| Variables | CVD | | | | | |
|  | coeff | se | t | P | LLCI | ULCI |
| Intervene | -0.25 | 0.12 | -2.08 | **＜0.05** | -0.48 | -0.015 |
| TyG-BMI | -0.33 | 0.18 | -1.82 | ＞0.05 | -0.68 | 0.026 |
| Gender | -0.55 | 0.25 | -2.23 | **＜0.05** | -1.03 | -0.067 |
| Int_1 | 0.10 | 0.055 | 1.82 | ＞0.05 | -0.008 | 0.21 |
| Int_2 | 0.16 | 0.077 | 2.08 | **＜0.05** | 0.0088 | 0.31 |
| Int_3 | 0.25 | 0.11 | 2.18 | **＜0.05** | 0.025 | 0.47 |
| Int_4 | -0.07 | 0.035 | -1.98 | **＜0.05** | -0.14 | -0.0008 |
| Int 1: Intervene × TyG-BMI; Int 2: Intervene × gender; Int 3: TyG-BMI × gender; Int 4: Intervene × TyG-BMI × gender. Controlled for age, educational level, marital status, total annual household income and ethnic. | | | | | | |

| Table S3 The moderation analysis between HRBs, TyG-BMI, age and CVD | | | | | | |
| --- | --- | --- | --- | --- | --- | --- |
| Variables | CVD | | | | | |
|  | coeff | se | t | P | LLCI | ULCI |
| Intervene | -0.05 | 0.047 | -1.12 | ＞0.05 | -0.15 | 0.04 |
| zMS | 0.088 | 0.049 | 1.80 | ＞0.05 | -0.008 | 0.18 |
| Gender | -0.078 | 0.097 | -0.80 | ＞0.05 | -0.27 | 0.11 |
| Int_1 | -0.027 | 0.016 | -1.72 | ＞0.05 | -0.058 | 0.004 |
| Int_2 | 0.025 | 0.03 | 0.83 | ＞0.05 | -0.034 | 0.08 |
| Int_3 | -0.07 | 0.033 | -2.16 | **＜0.05** | -0.14 | -0.0064 |
| Int_4 | 0.02 | 0.01 | 1.96 | **＜0.05** | 0.000 | 0.04 |
| Int 1: Intervene × TyG-BMI; Int 2: Intervene × gender; Int 3: TyG-BMI × gender; Int 4: Intervene × TyG-BMI × gender. Controlled for age, educational level, marital status, total annual household income and ethnic. | | | | | | |

| Table S4 Model characteristics for the conditional process analysis. | | | | | | |
| --- | --- | --- | --- | --- | --- | --- |
| Variables | CVH | | | CVD(sum) | | |
|  | B | t value | *P* value | B | t value | *P* value |
| Intervene | -0.038 | -1.79 | ＞0.05 | 0.013 | 2.25 | **＜0.05** |
| Gender | -0.23 | -0.45 | ＞0.05 | -0.22 | 0.99 | ＞0.05 |
| Intervene*Gender | 0.029 | 2.21 | **＜0.05** | -0.006 | -1.79 | ＞0.05 |
| CVH |  |  |  | -0.04 | -2.05 | **＜0.05** |
| CVH*Gender |  |  |  | 0.034 | 2.60 | **＜0.01** |
| R^2^ | 0.049 | | | 0.008 | | |
| F | 29.85 | | | 2.98 | | |
| Mediate variables: CVH, moderated variables: gender, independent variables: intervene, dependent variables: CVD. uncontrolled | | | | | | |

| Table S5 Bootstrapped conditional direct and indirect effects. | | | | | |
| --- | --- | --- | --- | --- | --- |
|  |  | | HRB clustering | | |
| Direct effect |  |  | Effect | SE | (LL,UL) |
|  | Predictor | SERF |  |  |  |
|  | Moderator (gender) | Male | 0.0065 | 0.0026 | 0.0014, 0.0116 |
|  |  | Female | 0.0001 | 0.0025 | -0.0048, 0.0049 |
| Indirect effect |  |  | Effect | SE | (LL,UL) |
|  | Predictor | HRB |  |  |  |
|  | Mediator (CVH) | Low | 0.0001 | 0.0001 | -0.0001, 0.0006 |
|  |  | High | 0.0005 | 0.0003 | 0.0001, 0.0013 |

| Table S6 Model characteristics for the conditional process analysis. | | | | | | |
| --- | --- | --- | --- | --- | --- | --- |
| Variables | CVH | | | CVD(sum) | | |
|  | B | t value | *P* value | B | t value | *P* value |
| Intervene | -0.062 | -2.83 | **＜0.01** | 0.0047 | 0.44 | ＞0.05 |
| HbA1c | -0.31 | -0.59 | ＞0.05 | -0.31 | 0.18 | ＞0.05 |
| Intervene*Gender | 0.035 | 2.60 | **＜0.01** | -0.001 | 0.79 | ＞0.05 |
| CVH |  |  |  | -0.029 | 0.14 | ＞0.05 |
| CVH*Gender |  |  |  | 0.026 | 1.96 | **＜0.05** |
| R^2^ | 0.057 | | | 0.005 | | |
| F | 35.25 | | | 1.79 | | |
| Mediate variables: CVH, moderated variables: HbA1c, independent variables: intervene, dependent variables: CVD. uncontrolled | | | | | | |

| Table S7 Bootstrapped conditional direct and indirect effects. | | | | | |
| --- | --- | --- | --- | --- | --- |
|  |  | | HRB clustering | | |
| Direct effect |  |  | Effect | SE | (LL,UL) |
|  | Predictor | SERF |  |  |  |
|  | Moderator (gender) | Male | 0.0037 | 0.0028 | -0.0017, 0.0091 |
|  |  | Female | 0.0027 | 0.0025 | -0.0022, 0.0076 |
| Indirect effect |  |  | Effect | SE | (LL,UL) |
|  | Predictor | HRB |  |  |  |
|  | Mediator (CVH) | Low | 0.0001 | 0.0003 | -0.0004, 0.0007 |
|  |  | High | 0.0002 | 0.0003 | -0.0002, 0.0009 |

| Table S8 The logistic regression analysis between behavioral and dietary interventions and metabolic index | | | | | | | | | |
| --- | --- | --- | --- | --- | --- | --- | --- | --- | --- |
| Variables | Exercise+diet | No Exercise+diet | Exercise+no diet | No Exercise+no diet |  | Exercise+diet | Exercise+no diet | No Exercise+diet | No Exercise+no diet |
| VSI |  |  |  |  |  |  |  |  |  |
| High | 1.0 | 1.28(0.99,1.66) | 1.17(0.61,2.27) | **1.42(1.004,1.99)*** |  | 1.0 | 1.12(0.57,2.20) | 1.09(0.83,1.43) | 1.35(0.94,1.93) |
| Medium | 1.0 | 1.19(0.93,1.53) | 1.06(0.55,2.05) | 1.14(0.80,1.61) |  | 1.0 | 1.02(0.52,2.00) | 1.05(0.81,1.37) | 1.09(0.76,1.56) |
| Low | 1.0 | 1.0 | 1.0 | 1.0 |  | 1.0 | 1.0 | 1.0 | 1.0 |
| TyG |  |  |  |  |  |  |  |  |  |
| High | 1.0 | **1.33(1.03,1.71)*** | 1.72(0.92,3.22) | 1.32(0.93,1.88) |  | 1.0 | 1.55(0.82,2.92) | 1.20(0.92,1.57) | 1.26(0.88,1.81) |
| Medium | 1.0 | 1.21(0.94,1.56) | 0.78(0.38,1.63) | 1.31(0.93,1.85) |  | 1.0 | 0.74(0.35,1.55) | 1.17(0.90,1.51) | 1.30(0.91,1.84) |
| Low | 1.0 | 1.0 | 1.0 | 1.0 |  | 1.0 | 1.0 | 1.0 | 1.0 |
| TG/HDL |  |  |  |  |  |  |  |  |  |
| High | 1.0 | 1.22(0.95,1.58) | 1.33(0.70,2.51) | 1.33(0.95,1.87) |  | 1.0 | 1.18(0.62,2.26) | 1.15(0.89,1.51) | 1.28(0.90,1.82) |
| Medium | 1.0 | 1.15(0.89,1.49) | 0.86(0.43,1.72) | 1.02(0.72,1.45) |  | 1.0 | 0.80(0.40,1.60) | 1.09(0.84,1.42) | 0.99(0.69,1.41) |
| Low | 1.0 | 1.0 | 1.0 | 1.0 |  | 1.0 | 1.0 | 1.0 | 1.0 |
| TyG-BMI |  |  |  |  |  |  |  |  |  |
| High | 1.0 | 1.13(0.88,1.46) | 1.69(0.88,3.26) | **1.40(1.001,1.97)*** |  | 1.0 | 1.51(0.78,2.93) | 1.0(0.76,1.30) | 1.29(0.91,1.83) |
| Medium | 1.0 | 1.26(0.98,1.62) | 1.16(0.57,2.34) | 1.13(0.79,1.60) |  | 1.0 | 1.09(0.54,2.21) | 1.18(0.91,1.53) | 1.08(0.75,1.54) |
| Low | 1.0 | 1.0 | 1.0 | 1.0 |  | 1.0 | 1.0 | 1.0 | 1.0 |
| TyG-WC |  |  |  |  |  |  |  |  |  |
| High | 1.0 | 1.12(0.87,1.45) | 1.85(0.93,3.67) | **1.40(1.001,1.96)*** |  | 1.0 | 1.65(0.82,3.31) | 1.07(0.82,1.40) | 1.31(0.93,1.86) |
| Medium | 1.0 | **1.38(1.07,1.77)*** | 1.63(0.81,3.31) | 1.14(0.81,1.63) |  | 1.0 | 1.55(0.77,3.15) | **1.35(1.04,1.75)*** | 1.14(0.80,1.63) |
| Low | 1.0 | 1.0 | 1.0 | 1.0 |  | 1.0 | 1.0 | 1.0 | 1.0 |
| zMS |  |  |  |  |  |  |  |  |  |
| High | 1.0 | 1.18(0.92,1.53) | 1.90(0.96,3.79) | **1.46(1.03,2.05)*** |  | 1.0 | 1.70(0.84,3.42) | 1.11(0.85,1.45) | 1.33(0.93,1.89) |
| Medium | 1.0 | 1.12(0.87,1.44) | 1.48(0.73,3.0) | 1.06(0.75,1.51) |  | 1.0 | 1.40(0.69,2.85) | 1.08(0.83,1.40) | 1.0(0.70,1.43) |
| Low | 1.0 | 1.0 | 1.0 | 1.0 |  | 1.0 | 1.0 | 1.0 | 1.0 |
| FBG |  |  |  |  |  |  |  |  |  |
| <6.1 | **0.56(0.37,0.83)**** | **0.25(0.07,0.82)*** | **0.46(0.30,0.69)**** | 1.0 |  | **0.55(0.36,0.83)**** | **0.23(0.07,0.78)**** | **0.45(0.30,0.68)*** | 1.0 |
| 6.1-6.99 | 1.43(0.91,2.24) | 0.43(0.13,1.46) | 1.18(0.75,1.87) | 1.0 |  | 1.46(0.92,2.32) | 0.47(0.14,1.61) | 1.23(0.77,1.95) | 1.0 |
| ≥7.0 | 1.0 | 1.0 | 1.0 | 1.0 |  | 1.0 | 1.0 | 1.0 | 1.0 |

**Figure legends**


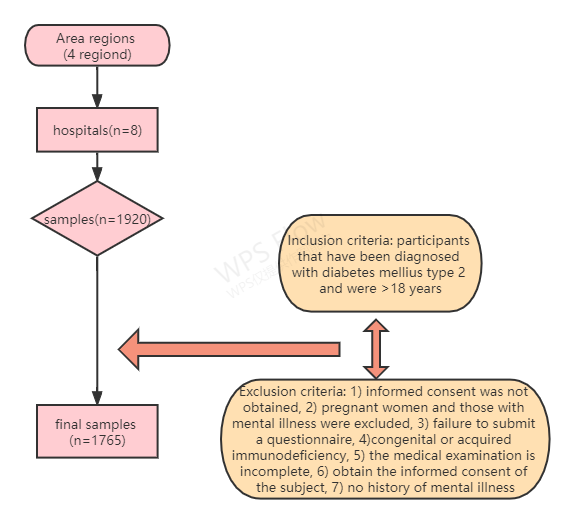


**Fig. S1 Flow chart of participants in the study**


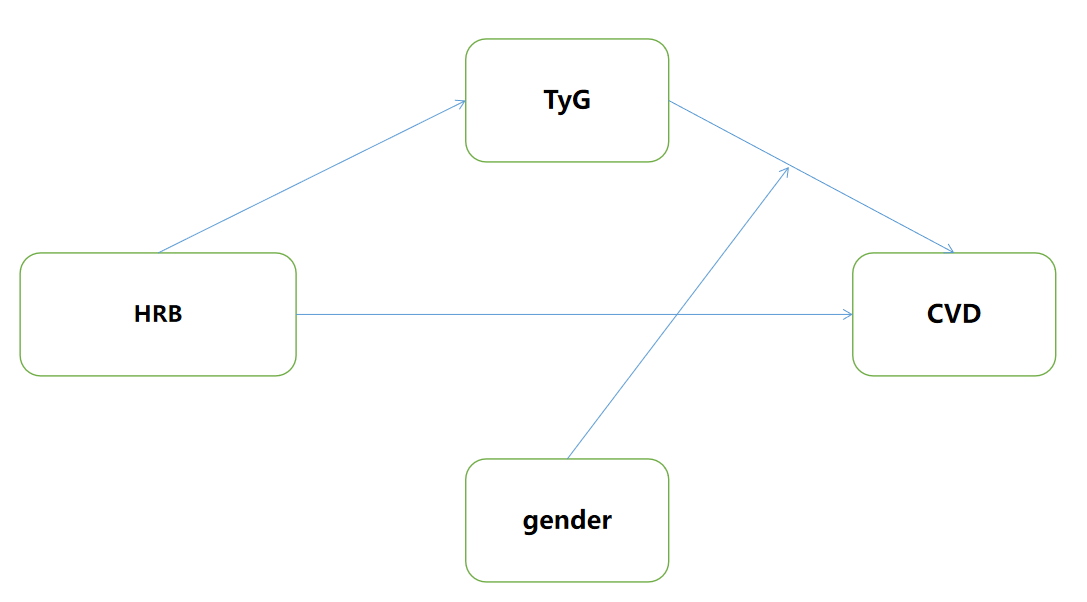


**Fig S2 Mediating moderation analysis**


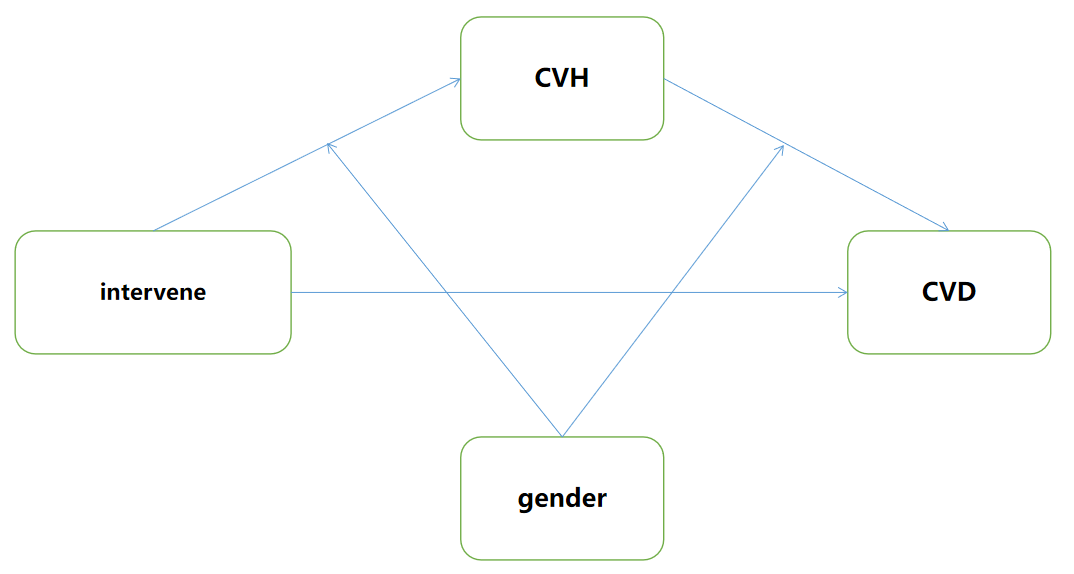


**Fig S3 Medial moderation analysis**
